# High Potassium Diet Rich in Spices and Herbs-Salt Substitution (HPSH-SS) for Blood Pressure Reduction in Older Adults: Protocol for Diet Concept and Randomized Controlled Trial

**DOI:** 10.2196/56869

**Published:** 2024-10-29

**Authors:** Farapti Farapti, Sheila Amara Putri, Annisaa Wulida Furqonia, Purwo Sri Rejeki, Muhammad Miftahussurur

**Affiliations:** 1 Department of Nutrition Faculty of Public Health Universitas Airlangga Surabaya Indonesia; 2 Doctoral Program of Medical Science Faculty of Medicine Universitas Airlangga Surabaya Indonesia; 3 Government of Sobrah Village Wungu District Madiun Regency Indonesia; 4 Department of Physiology Faculty of Medicine Universitas Airlangga Surabaya Indonesia; 5 Gastroentero-Hepatology Division Department of Internal Medicine Dr Soetomo Teaching Hospital Surabaya Indonesia; 6 Institute of Tropical Disease Universitas Airlangga Surabaya Indonesia

**Keywords:** sodium, potassium, spices and herbs, blood pressure, hypertension, elderly, vascular, kidney, gerontology, aging, protocol study, dietary, phytochemical, anti-hypertensive, Indonesia, molecular mechanism, control group, oxidative stress

## Abstract

**Background:**

Hypertension increases with age, often due to high sodium (Na) and low potassium (K) intake. Reducing salt and increasing K intake is challenging, especially for older adults due to taste preferences. Culinary herbs and spices, rich in K, offer a potential solution. The High Potassium Diet Rich in Spices and Herbs-Salt Substitution (HPSH-SS) diet has not yet been studied for its effectiveness in lowering blood pressure.

**Objective:**

This study aims to create an HPSH-SS diet, analyze its effects on blood pressure in older adults, and study the molecular mechanism occurring in the kidneys and blood vessels influenced by this diet.

**Methods:**

This study consists of 2 phases. The first phase involved formulating and assessing the HPSH-SS diet tailored for older adults. The intervention group (IG) received a diet of 1800 kcal/day, with 3500 mg K and 1500 mg Na, while the control group (CG) received 1500 mg K and 2000 mg Na. The diet was administered for 14 days and standardized using the NutriSurvey program and biochemistry analysis by atomic absorbance spectrophotometry (AAS). The second phase was a 14-day parallel randomized controlled trial (RCT) with the older adult participants divided into IG and CG. Primary outcomes included blood pressure; serum potassium; aldosterone; F2 isoprostane; nitric oxide plasma levels; and urine analysis of Na, K, and the Na/K ratio. Confounding variables were controlled through randomization and stratified analysis.

**Results:**

The menu formulation and organoleptic assessment of the HPSH-SS diet began in mid-2022 and was approved by the Ethics Committee of the Faculty of Public Health at Universitas Airlangga (78/EA/KEPK/2022) on May 11, 2022. The diet was standardized to achieve daily nutritional values of 1800 kcal energy, 3500 mg K, and 1500 mg Na. K and Na contents were analyzed using AAS from several participants’ spice diet menus. Recruitment for the RCT started in March 2023, with approval from the Health Research Ethics Committee Universitas Airlangga School of Medicine, Surabaya (35/EC/KEPK/FKUA/2023). The study was registered from February 9, 2023, to February 9, 2024. Between March and June 2023, 64 participants were recruited, with 32 participants in the IG and CG. The intervention and data collection will take place over 1 year. Data management is in progress, and data analysis is yet to be performed.

**Conclusions:**

This RCT protocol hypothesizes that the diet will increase serum K, plasma aldosterone, and nitric oxide levels; decrease plasma F2 isoprostane; increase urinary Na and K levels; lower the urinary Na/K ratio; and reduce systolic and diastolic blood pressure. If effective, it will offer valuable insights into dietary strategies for blood pressure regulation in older adults.

**International Registered Report Identifier (IRRID):**

DERR1-10.2196/56869

## Introduction

Hypertension is a major public health problem worldwide, and its prevalence continues to rise with age. The prevalence of hypertension is predicted to increase around 60% by 2025 with nearly 1.5 million deaths, accounting for 9.4% of total deaths annually [1,2]. Both modifiable and nonmodifiable risk factors for developing hypertension are considered, with a focus on diet modifications as a modifiable risk factor. The risk factors for hypertension are closely related to dietary habits, particularly excess dietary salt and potassium (K) deficiency [3]. Population studies reported that most populations around the world consume less than the recommended intake of K, while unfavorably high sodium (Na) intake remains prevalent globally [4-6]. It is widely known that excessive Na consumption and insufficient K intake play important roles in the pathogenesis of hypertension and are more strongly associated with blood pressure than either Na or K alone [7,8]. 

Nonpharmacological therapy is the primary approach for managing hypertension, consisting of lifestyle changes and dietary patterns aimed at lowering blood pressure and controlling risk factors and comorbidities. Salt reduction strategies have become a cornerstone program in various countries, including Indonesia, to reduce the incidence of hypertension and cardiovascular diseases. Various salt reduction programs have been proven to be low-cost and effective, and some countries have successfully reduced salt consumption in their populations. However, Na or salt consumption among the global population still exceeds the World Health Organization (WHO) recommendation of 5 g per day [5,9-14]. A low-cost strategy for managing hypertension is to reduce salt in food. However, this is not always easy due to issues with taste and food acceptance, especially since older adults often have a high salty taste threshold [15-17]. This can lead to decreased appetite, potentially resulting in long-term malnutrition and deteriorating health status [15,18]. Another strategy is salt substitution. Recent studies have found a way to replace salt with spices and yielded good results regarding taste acceptance [19]. Some practices involve replacing sodium chloride (NaCl) with alternatives like potassium chloride (KCl) or calcium chloride (CaCl); however, these substitutes can have drawbacks, including a bitter taste and potential toxicity risks [16,20]. Substitution with monosodium glutamate (MSG) has also been reported. While MSG can replace the function of salt without compromising taste acceptance, its use in large quantities has been associated with various side effects [21,22]. Recent studies have investigated substituting salt with herbs and spices, yielding promising results in taste acceptance; however, adding spices may alter the taste and aroma of food, which may not be universally accepted. Studies on salt substitution with spice blends remain limited and are generally conducted on a single type of food [17,23].

Programs for increasing K intake at the population level are still rare and not progressive. K is a nutrient closely related to diet quality, although the cost of the diet may inhibit its intake [24,25]. It is recommended that K be consumed through food due to its safety and lack of an upper intake limit. Therefore, presenting food sources of K that are affordable and commonly used is an important requirement [5,24,26]. Herbs and spices with their phytochemical components have been widely studied as antihypertensives [27,28]. However, the relation to K content in spices, which acts as an antihypertensive agent, has not been widely studied. A high K and low Na diet, which includes 4 to 5 servings of fruits and vegetables and is low in fat, is known as the Dietary Approaches to Stop Hypertension (DASH) diet [29,30]. The benefits of the DASH diet have been recognized by general dietary guidelines from the US National Heart, Lung, and Blood Institute (NHLBI); the US Department of Agriculture (USDA); the International Diabetes Federation (IDF); and the European Association for the Study of Diabetes (EASD) [31-33]. However, the DASH diet has not yet been fully applied and studied in Indonesia. Indonesia is the largest producer of herbs and spices in the world, which can easily be found in traditional markets, supermarkets, and even in peoples’ homes [27].

The kidney is the main regulator of Na and K in the blood. High K intake stimulates gastrointestinal signaling, specifically enteric K sensors, which increases plasma K levels and aldosterone directly. Aldosterone stimulates the aldosterone-sensitive distal nephron (ASDN) and the renal outer medullary potassium channel (ROMK), which triggers a decrease in salt and water absorption, causing diuresis and natriuresis. High K intake stimulates kaliuresis, natriuresis, and diuresis by increasing plasma K from both aldosterone and nonaldosterone pathways. This occurs via the inactivation of phosphorylation of the natrium-chloride cotransporter (NCC) and increased ASDN and ROMK [34]. Increased K intake inhibits free radical formation from endothelial dysfunction and vascular smooth muscle cell proliferation. Increased K also inhibits platelet aggregation and arterial thrombosis, conditions often associated with platelet oxidative stress in older adults. Consequently, elevated K levels decrease urine and plasma isoprostane F2 levels [35]. Additionally, increased K plasma levels improve endothelial cell stiffness and increase nitric oxide (NO) release through the mechanism of cortical actin depolarization by cytochalasin [36,37].

Indonesia is the largest producer of herbs and spices in the world, and their consumption among older adults is common. Spices and herbs are proven to have high K content and can be applied to salt substitution methods. However, a healthy diet specifically designed with these components, known as the High Potassium Diet Rich in Spices and Herbs-Salt Substitution (HPSH-SS), has not yet been established. Since population-level programs to increase K intake, particularly among older adults, are still rare and widely implemented, this study aims to develop and evaluate the HPSH-SS diet. This study will analyze the effect of this diet on blood pressure in older adults and investigate the molecular mechanisms occurring in the kidneys and blood vessels influenced by the diet. The data generated will provide valuable information in developing new approaches to managing blood pressure in older adults.

## Methods

### Study Overview

Data collection was carried out at a government institutional setting in Surabaya, Indonesia, namely Griya Wreda Surabaya nursing home. This study enrolled older adult residents at the nursing home in 2023 as participants. The research implementation was divided into 3 periods: the pretreatment period (day –10 to day 0), the treatment period (day 1 to day 14), and the posttreatment period (day 15). A parallel randomized controlled trial (RCT) intervention study compared the intervention group (IG), who received a low-salt, high-K spice-enriched diet (3500 mg K and 1500 mg Na per day), with the control group (CG), who received a control diet (1700 mg K and 2000 mg Na per day) for 14 consecutive days.

This study involved almost all workers or staff members at the nursing home, including cooks, nurses, and nutrition experts during data collection. Due to institutional regulations, we were not permitted to alter the daily intake provided by the care facility. As a result, we had to adjust the diet concept for this study. The initial step involved observing all menus and food compositions of the older adults’ daily intake. We then arranged and adjusted the diet to fit our study design: the IG received a diet with 3500 mg of K and 1500 mg of Na per day, while the CG received a diet with 1500 mg of potassium and 2000 mg of sodium per day. We specifically recorded the composition of spices and herbs in their menus and found that commercial seasoning packets were commonly used in main courses, such as side dishes and vegetables.

Cooks in the institutional setting, as meal providers, played a crucial role in facilitating the older adults’ daily food intake. They cooked daily menus as usual, the same for both groups. They received guidance and information about their involvement in this study. During the 14-day intervention period, cooks were instructed to use only commercial spices provided by the institution without adding any additional seasonings. They were also asked to reduce the salt content for the older adults’ meals, particularly those in the IG. Nurses were responsible for accompanying the older adults in their daily activities to ensure their health conditions remained optimal. In this study, nurses were involved in measuring blood pressure, collecting blood samples, collecting 24-hour urine samples, and recording any health complaints from the participants during the study period. Involving dietitians as part of the research team in this study was crucial, as there were no dietitians in the nursing home. The dietitians monitored and evaluated the older adults’ dietary intake. They observed the entire food preparation process, especially cooking, and recorded all ingredients used, including seasonings and added salt. Providing the intervention and control menus and evaluating the overall food leftovers, including main meals and intervention menus from the participants for 14 consecutive days, were the dietitians’ main tasks during the intervention period.

### Study Design

#### Description

This study consisted of 2 phases. The first step involved trials of menu formulation and organoleptic assessment of the HPSH-SS diet concept, which began in mid-2022. The next phase is the RCT, with recruitment starting in March 2023. The intervention and data collection will take place over 1 year.

#### Phase 1: Trials of Menu Formulation and Organoleptic Assessment

Developing the HPSH-SS diet involved trials of menu formulation and organoleptic assessment, which began in mid-2022. We did not provide the daily intake entirely because the nursing home did not allow changes to its usual menu. Instead, we adjusted the existing menu to align with the HPSH-SS diet concept by arranging a low-salt, high-K spice-enriched diet (3500 mg K and 1500 mg Na per day) and a control diet (1500 mg K and 2000 mg Na per day). The initial step was to observe the older adults’ daily menus. The nutritional composition of the foods was calculated using the NutriSurvey software (EBISpro).

Based on the nutritional values of the older adults’ menu, the next step was to design intervention and control menus to achieve nutritional values that aligned with this study’s diet concept. Both the IG and CG were provided with the same energy intake of approximately 1800 kcal. However, Na and K were the main variables in this spice-enriched diet, and their roles were specifically examined. The IG received 3500 mg of K and 1500 mg of Na, while the CG received 1500 mg of K and 2000 mg of Na. Therefore, adjustments were made to the daily dietary menus of the older adults in the nursing home to meet the designed intervention’s nutritional targets.

**Figure 1:**
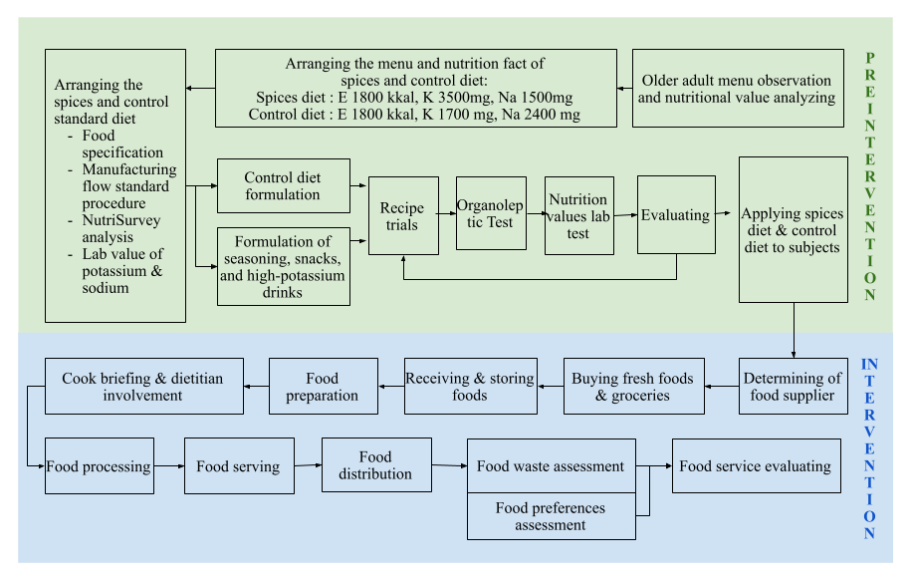
Flowchart of the High Potassium Diet Rich in Spices and Herbs-Salt Substitution (HPSH-SS) intervention and monitoring evaluation.

For each intervention menu recipe, a detailed report on food ingredient specifications and standard operational procedures (SOPs) for the production process was prepared beforehand. The next step involved conducting trials and organoleptic taste evaluations for taste acceptance by a limited panel of testers and participants, followed by biochemical laboratory examinations for K and Na nutritional values. The entire process sequence is described in Figure 1.

#### Phase 2: RCT Study

The RCT study was implemented with recruitment commencing in March 2023 until the end of 2023. This study is a parallel randomized controlled feeding trial testing the effect of 2 dietary patterns on blood pressure by analyzing serum K levels, plasma aldosterone, plasma isoprostane F2, plasma NO, urinary Na, urinary K, and urinary Na/K ratio changes in the older adults with hypertension for 14 consecutive days. Both IG and CG were provided with the same energy intake of approximately 1800 kcal. The IG received 3500 mg of K and 1500 mg of Na, while the CG received 1700 mg of K and 2000 mg of Na.

#### Study Population and Participants

The study population consisted of older adults registered as residents in an institutional nursing home in Surabaya, Indonesia. The sample size for each group was set at a minimum of 28 participants. The sample size was calculated following the formula by Lachenbruch et al [38]. Based on SD calculations by Geleijnse et al [39], the sample size yielded a minimum of 25 participants for each group. When accounting for a 10% dropout rate, the minimal sample size for each group was 28 individuals, resulting in a total sample size of 56 participants for this study. The inclusion criteria were older adults aged ≥60 years residing in the nursing who were active and not bedridden and those without infectious disease, taste disorders, or dementia. Additionally, participants were required to have blood pressure measurements indicating a systolic blood pressure (SBP) ≥130 mmHg and/or diastolic blood pressure (DBP) ≥85 mmHg. The exclusion criteria involved individuals with impaired kidney function (creatinine serum levels >1.2 mmol/L), uncontrolled diabetes mellitus (fasting blood sugar >126 mg/dL), obesity (BMI ≥30 kg/m^2^), active smokers, and those with memory impairment and depressive conditions. Additionally, participants declining continued participation, requiring intensive hospital care, or experiencing persistent vomiting and diarrhea were excluded from the intervention.

### Study Variables

This study included independent, dependent, mediating, controlling, and confounding variables. Independent variables were the HPSH-SS diet and the control diet. Dependent variables included SBP and DBP. Mediating variables were serum K level, plasma aldosterone, urine Na, urine K, plasma F2 isoprostane, and plasma NO. Controlling variables included the duration of the intervention, the treatment timing and method, the evaluation timing and method, participant criteria, laboratory examination of outcome results, equal energy content of both diets, and sources of the food ingredients and commercial seasonings containing spices and herbs used for both groups, as well as cooks involved in both the food preparation and presentation. Finally, the confounding variables consisted of age, gender, consumption of antihypertensive medications, amount of diet consumed during the intervention period, occurrence of mild nausea and vomiting during the intervention period, and other metabolic diseases such as dyslipidemia and hyperuricemia. The randomization process was used to control the confounding variables (age, gender, and consumption of antihypertensive drugs) between the 2 groups to ensure that the participant characteristics were evenly distributed between both groups.

### Operational Framework

**Figure 2:**
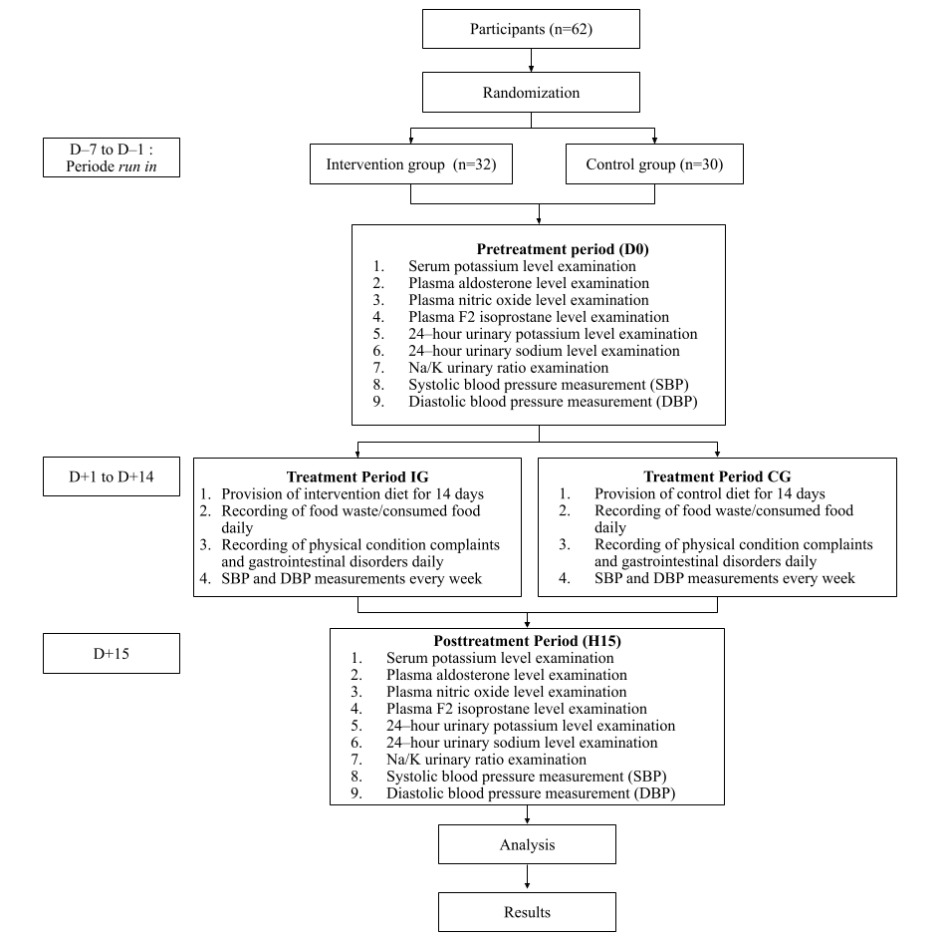
Operational framework. CG: control group; IG: intervention group.

The research implementation was divided into three periods as shown in the operational framework in Figure 2: (1) the pretreatment period, (2) the treatment period, and (3) the posttreatment period. The pretreatment period (day –10 to day 0) consisted of giving information to the participants and explaining the study’s purpose and benefits. Then, interviews were conducted to obtain baseline data (age, medical history, and medication consumption), anthropometric measurements (including body weight and height), and blood pressure measurements. Moreover, screening for kidney function and serum K levels was performed. Participants who met the research criteria were divided into 2 groups, namely IG and CG, through randomization. Starting from day 7, the participants entered a 7-day run-in period to standardize food intake and physical activity. The older adults were educated not to change their usual eating patterns or activities. Both groups consumed meals provided only by the institution. On day –1, participants were informed and briefed on the treatment they would receive, including receiving a new menu to be consumed for 14 consecutive days and the evaluation and various examinations that would be conducted. Finally, on day 0, SBP and DBP measurements were taken, and serum K levels, plasma aldosterone, urine Na and K levels, plasma F2 isoprostane levels, and plasma NO were examined.

The treatment period (day +1 to day +14) consisted of the participants consuming the diet every day for 14 days consecutively. Every mealtime, participants were asked and assessed about taste acceptance and food waste for the food served. The older adults were informed that uneaten food should not be discarded and should be kept on the plate for recording by the research team. On the morning of day +8, SBP and DBP measurements were conducted by trained nurses following standardized measurement procedures. During the intervention, any complaints of health disturbances experienced by the participants, especially those related to digestive problems such as nausea, vomiting, or diarrhea, were noted and addressed by providing medication to reduce and treat the issues. Finally, on the posttreatment period (day +15), SBP and DBP measurements were taken again, and serum K levels, plasma aldosterone, urine Na and K levels, plasma F2 isoprostane levels, and plasma NO were examined.

This study sought to determine whether a high potassium spice diet and salt substitution in older adults with a history of hypertension can increase serum potassium levels, increase plasma aldosterone levels, increase plasma nitric NO levels, decrease plasma F2 isoprostane levels, increase urinary Na and K levels, decrease the urinary Na/K ratio, and decrease SBP and DBP.

### Statistical Analysis

All statistical calculations were conducted with SPSS software (version 21.0; IBM Corp), and *P*<.05 was considered significant. The Shapiro-Wilk test was used to check normality data. In normal distributions for continuous variables, mean and SD were applied, while in abnormal distributions, the median and minimum to maximum were used. Data classified as categorical variables were summarized as numbers and percentages. Bivariate analysis was carried out to analyze the different outcomes between the 2 groups (IG and CG) using the *t* test or the Mann-Whitney *U* test. To compare ratio data between the pre- and posttest, a paired *t* test or Wilcoxon test was used.

### Ethical Considerations

In mid-2022, trials began on menu formulation and organoleptic assessments for the HPSH-SS diet concept. Ethics approval was granted by the Ethics Committee of the Faculty of Public Health, Universitas Airlangga (78/EA/KEPK/ 2022) on May 11, 2022. The application of the RCT study started in March 2023. Ethical approval was obtained from the Health Research Ethics Committee at Universitas Airlangga School of Medicine, Surabaya, Indonesia (35/EC/KEPK/FKUA/2023) for the study titled “The Mechanism of Lowering Blood Pressure in Older adults with Diet of Spice and Herb High Potassium and Salt Substitution,” registered on February 9, 2023. All procedures performed in this study involving human participants were conducted in accordance with the ethical standards outlined by the committee. The data collection procedure was initiated by seeking approval from the head of the institutional nursing home and the participants. The research commenced with a preliminary socialization process. The participants sign an informed consent after receiving a detailed and clear explanation about the study, including the research title, the benefits of participation, and the research procedures. This study was submitted for registration in clinicaltrials.gov at the end data collection and analysis.

## Results

### The HPSH-SS Diet Concept

The initial step before arranging diet menus was observing the older adults’ daily menu in the home and analyzing its nutritional value. Table 1 shows the nutritional values of the master menu providing approximately 1321 kcal of energy, which met around 73% of the energy requirement, 1661 mg of Na, and 130 mg of K, accounting for only about 30% of the recommended intake.

**Table 1:** Nutritional values of the master menu at the Griya Werdha Surabaya nursing home.

Nutritional values	Energy (kcal)	Protein (g)	Fat (g)	Carb (g)	Fiber (g)	Na^a^ (mg)	K^b^ (mg)	Calcium (mg)
Daily average, mean (SD)	1321 (109.59)	47 (5.79)	53 (10.18)	168 (18.94)	7 (1.82)	1661 (363.9)	1305 (211.39)	289 (100.67)
RDA^c^ and fulfillment, n (%)	1800 (73)	65 (73)	50 (106)	275 (61)	25 (28)	1500 (111)	4700 (28)	1200 (24)

^a^Na: sodium.

^b^K: potassium.

^c^RDA: recommended dietary allowance.

Considering the nutritional values of the older adults’ menu, the next step was to design intervention and control menus to achieve nutritional values according to this study’s diet concept. The energy content was arranged to meet the nutritional needs of the older adults, consisting of 1800 kcal/day, so both groups (IG and CG) were provided with the same energy intake. The IG received 3500 mg of K and 1500 mg of Na, while the CG received 1500 mg of K and 2000 mg of Na daily. Therefore, adjustments were needed in the daily dietary menu of the older adults at the nursing home to align with the nutritional values of the designed intervention.

Since we were not permitted to change the usual menus provided by the nursing home, the main breakfast, lunch, and dinner menus were the same as usual; however, we added a combination of fried onion and garlic and reduced salt in the main meals. Furthermore, to fulfill the K and Na nutritional requirements of both diets, we added snacks and a beverage. Additional food menus for both groups were created with different nutritional contents (Table 2).

K and Na were examined using atomic absorbance spectrophotometry (AAS) from the additional foods in both groups. Tables 3 and 4 show the nutrient compositions of the HPSH-SS menus and the control menus, respectively.

**Table 2:** Additional foods in the intervention and control menus.

Mealtime	HPSH-SS^a^ menus	Control menus
7 AM (breakfast)	Herbal tea	Teabag
10 AM (morning snack)	Cinnamon spinach pudding	Sweet milk pudding
2 PM (afternoon snack)	Mung bean Kampferia galanga	Coconut milk dawet
7 PM (evening snack)	Spices potato schotel	Tofu noodle schotel
Additional treatments	Onions (10 g) and garlic (5 g) in every main meal and salt amount reduced when cooking	N/A^b^

^a^HPSH-SS^:^ High Potassium Diet Rich in Spices and Herbs-Salt Substitution.

^b^N/A: not applicable.

**Table 3:** Nutrient composition of the HPSH-SS^a^ menus.

Menu	Energy (kcal) (N=548.4)	K^b^ (mg) (N=2193.8)	Na^c^ (mg) (N=152.5)
Ginger tea	79	402.8	3.4
Cinnamon spinach pudding	64.4	336	15.7
Mung bean kaempferia galanga	182.1	600	21.3
Spiced potato schotel	147.9	780	62.2
Fried onion and garlic	7	75	50
Master menu of the older adults	1321	1661	1305
Total intake	1869	3555	1563

^a^HPSH-SS^:^ High Potassium Diet Rich in Spices and Herbs-Salt Substitution.

^b^Na: sodium.

^c^K: potassium.

**Table 4:** Nutrient composition of the control menus.

Menu	Energy (kcal) (N=601.2)	K^a^ (mg) (N=188.8)	Na^b^ (mg) (N=433.3)
Tea (400 ml)	109	0.6	12
Milk pudding	66.2	11.4	2.7
Dawet coconut milk (400 ml)	286.5	87.2	24.1
Tofu schotel (80 g)	139.5	89.6	394.5
Total intake	1922	1494	2094

^a^Na: sodium.

^b^K: potassium.

**Figure 3:**
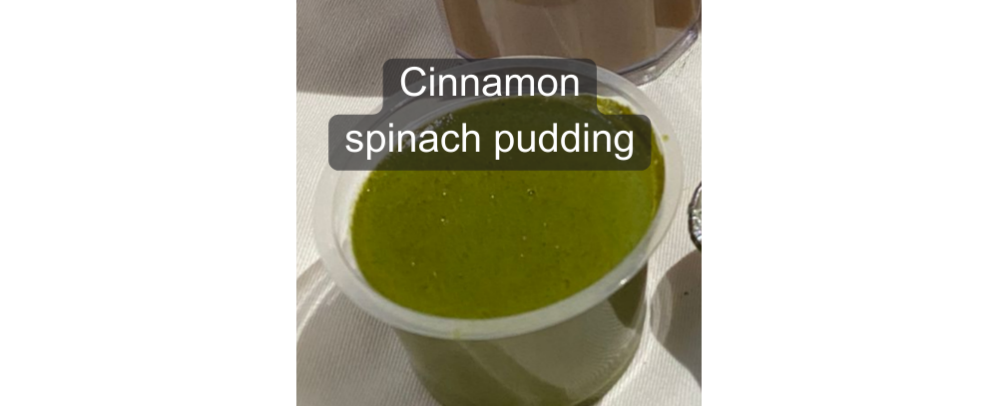
Cinnamon spinach pudding.

Equal energy content was maintained in both diets. Food ingredient sources, commercial seasonings containing spices and herbs used in both groups, and food preparation and presentation were the controlled variables. All menu recipes followed SOPs for the production process, including the evaluation of taste acceptance and food taste. Figure 3 shows the composition of one of the menu items, spinach pudding, which was a morning snack in the HPSH-SS diet consumed by the IG every day during the intervention period. It contained the following ingredients and specifications: 125 g agar-agar, 25 g Nutrijell, 30 g spinach, 35 g cinnamon, 75 g milk, 14 g sugar, and 75 ml water. We applied spinach and cinnamon as K-source foods. Moreover, cinnamon was chosen as a spice with a high K content.

### RCT Study

The recruitment of randomized controlled trials started in March 2023. The study was approved by the Health Research Ethics Committee Universitas Airlangga School of Medicine, Surabaya (35/EC/KEPK/FKUA/2023), and all participants provided written consent. The study was registered for the period between February 9, 2023, and February 9, 2024. Intervention and data collection will take place for 1 year. During the beginning of the research period (March-June 2023), 64 participants had already been recruited for the study, with 32 assigned to the IG and CG. Data management and analysis are still in progress, so data analysis has not yet been performed. This study was funded by the Indonesian Directorate of Research, Technology, and Community Service and the Universitas Airlangga for Doctoral Dissertation Research Grant 2023.

## Discussion

A high prevalence of hypertension in older adults persists as a major public health issue worldwide. Excessive Na or salt consumption and low intake of K play a vital role in the pathogenesis of hypertension and cardiovascular disease [7,40]. Comprehensive strategies are needed to simultaneously reduce salt and increase K intake, as both are essential nutrients that work complementarily at the cellular and are more strongly associated with blood pressure than either Na or K alone [41]. This study aims to create a diet containing high K and low Na intake and analyze the effects of this diet on blood pressure in older adults. This parallel RCT intervention study compares an IG receiving a high-K spice diet (3500 mg K and 1500 mg Na per day) with a CG receiving a control diet (1500 mg K and 2000 mg Na per day) for 14 consecutive days. This adhered to SOPs for the production process of the diet, including the evaluation process of taste acceptance and food waste for every food menu eaten by the older adults. This RCT will test the hypothesis that the HPSH-SS diet is effective in lowering blood pressure by examining some markers of vascular and kidney in older adults.

Na or salt consumption reduction has become a major health program in various countries worldwide to reduce the incidence of hypertension and cardiovascular diseases [42]. Epidemiological studies highlight excessively high dietary intake of sodium at the community level in most parts of the world [42]. Several countries have implemented salt-reduction strategy programs and have successfully limited dietary salt in their populations [10,43]. However, in older adults, this can be challenging due to issues related to high salt taste threshold and taste preference, which can lead to overall reduced intake [44]. According to WHO recommendations, total Na intake should not exceed 2 g/day, and extremely low Na consumption is not recommended, as both too low and too high intakes potentially increase the risk of mortality, especially for older adults [45,46]. Therefore, this study provided Na intervention at 1500 mg for the IG and 2000 mg for the CG. Our analysis of Na intake from the menus of the older adults in the nursing home indicated that their Na intake was not too high, averaging 1661 mg and meeting 111% of the recommended dietary allowance (RDA). Therefore, we reduced salt in the cooking process to 1500 mg for the IG. In contrast, the control menus allowed for an additional Na intake, increasing to 2000 mg from other food sources. We did not lower Na intake below 1500 mg because reducing salt intake too much can affect taste and lead to deficiencies and undernutrition in older adults over time [47,48]. Moreover, providing Na intake of more than 2000 mg could have harmful effects on older adults and is inappropriate for individuals with high blood pressure [49,50].

Most populations consume less than the requirement of K and still fail to reach the recommended levels, emphasizing the urgent need for dietary interventions. Unlike salt-reduction strategy programs that are low cost and effective [6], increasing K intake and achieving the recommended daily K intake of 4700 mg is challenging, particularly for low socioeconomic groups, as K-rich foods such as fruits, vegetables, legumes, and dairy products are associated with higher food costs [19,24]. Low K intake generally occurs due to low fruit and vegetable intake. A systematic review study showed almost all Indonesian people consume inadequate amounts of fruits and vegetables [51]. Additionally, previous studies have shown that K intake is associated with nutritional quality, so increasing it will improve diet quality and reduce blood pressure [24]. Specifically, for K intake, we did not adhere to the Indonesian RDA of 4700 mg per day because the K intake among older adults in the nursing home was only 1300 mg, or 28% of the daily requirement [51]. Instead, we administered 3500 mg of K in this study, which is in line with the WHO recommendation for a minimum daily K intake of 3500 mg, preferably from food sources rather than supplements [6].

This study provided Na intervention at 1500 mg for the IG and 2000 mg for the CG. An Na/K ratio of 1 is considered beneficial for health; in this study, the Na/K ratio was much above the recommended level. Finding effective ways to lower this ratio in the population by reducing Na consumption while promoting dietary K consumption is important [4,7]. Our study showed that the Na/K ratio in the HPSH-SS diet was 1500/3500 mg (less than 1), while in the control diet, it was 2000/1500 mg (more than 1). Interventions involving K intake and Na restriction have been carried out both through supplementation and food or dietary intake. High-K and low-Na diets, including 4 to 5 servings of vegetables and fruits per day, and low-fat DASH diets, have been proven effective in reducing blood pressure [29]. DASH diets with varying K contents and Na-restricted diets have been studied. However, the development of a DASH diet with the main addition of spices has not been reported. Since spices as a source of K have also been less studied, a 2023 study by Singh [52] examined the K, Na, and their ratio in 45 commercial cultivars of onions in India. The results of this study demonstrated that onions are a potential source of K and low Na. Strategies involving salt reduction face challenges, particularly regarding taste acceptance, especially among older adults. Replacing salt with spices has shown potential in addressing taste acceptance issues while benefiting blood pressure management.

A recent study by Farapti et al [53] demonstrated using herbs and spices is an effective method for salt substitution in food and is acceptable in terms of taste preference and taste acceptance by older adults. However, an implementation study of a spice diet in daily food menus for reducing blood pressure has not been reported, and it is the novel aspect of our study. Nevertheless, the detailed mechanism of reducing blood pressure in older participants is complicated. This study only evaluated the effectiveness of HPSH-SS diet on lowering blood pressure by vascular and kidney mechanisms, not including sympathetic nerves that might explain the comprehensive mechanism of reducing blood pressure. The result of this study might not be applicable to the general population, particularly older adults with impaired kidney function and other diseases.

Indonesia is the world’s largest producer of spices and herbs with a contribution of around 21.06% of the total world market [54]. Given the high consumption of spices among older adults in Indonesia, the HPSH-SS diet is expected to be well accepted. This study introduced K-rich food sources that are nutritionally high quality, affordably priced, readily available, and easy to process daily. Thus, this diet can be applied to fulfill the daily K intake. Additionally, this study presents innovative functional foods containing spices and herbs that are high in K for lowering BP. If proven effective, our findings could provide valuable insights into dietary strategies for blood pressure regulation, particularly for older populations, and can contribute to improving their overall health status.

## Abbreviations

AAS: atomic absorption spectrometry
ASDN: aldosterone-sensitive distal nephron
CaCl: calcium chloride
CG: control group
DASH: Dietary Approaches to Stop Hypertension
DPB: diastolic blood pressure
EASD: European Association for the Study of Diabetes
HPSH-SS: High Potassium Diet Rich in Spices and Herbs-Salt Substitution
IDF: International Diabetes Federation
IG: intervention group
K: kalium (potassium)
KCl: potassium chloride
MSG: monosodium glutamate
Na: natrium (sodium)
NaCl: sodium chloride
NCC: natrium-chloride cotransporter
NHLBI: National Heart, Lung, and Blood Institute
NO: nitric oxide
ROMK: renal outer medullary potassium channel
SBP: systolic blood pressure
SOP: standard operational procedure
USDA: United States Department of Agriculture
WHO: World Health Organization

## Data Availability

All data that will be used or analyzed in the study will be supplied upon reasonable request. Data supporting the findings of this study are included in the manuscript. Personal data are not publicly accessible due to ethical considerations, but they can be obtained from the corresponding author upon request.
